# Incidence of candidemia and prevalence of azole-resistant candidemia at a tertiary South African hospital – A retrospective laboratory analysis 2016–2020

**DOI:** 10.4102/sajid.v37i1.326

**Published:** 2022-02-15

**Authors:** Vindana Chibabhai

**Affiliations:** 1 Department of Clinical Microbiology and Infectious Diseases, Faculty of Health Sciences, University of the Witwatersrand, Johannesburg, South Africa; 2 Clinical Microbiology Laboratory, Charlotte Maxeke Johannesburg Academic Hospital, National Health Laboratory Service, Johannesburg, South Africa

**Keywords:** candidemia, incidence, antifungal resistance, invasive candidiasis, antifungal stewardship, *Candida auris*, *Candida parapsilosis*, surveillance

## Abstract

**Background:**

Candidemia is associated with high morbidity and mortality. The epidemiology of candidemia has changed globally over the past 20 years. South African surveillance demonstrated a shift in epidemiology from *Candida albicans* to non-albicans species including *Candida parapsilosis* and *Candida auris*. Hospital-level candidemia incidence from South Africa has not been reported previously.

**Methods:**

We performed a retrospective laboratory-based analysis of blood cultures with confirmed causative agents of candidemia. Ward type, department, gender and admission to critical care units were captured. Data were analysed in Microsoft Excel, Statistical Package for the Social Sciences (SPSS) and Epitools.

**Results:**

The incidence of candidemia during the study period was 2.87 per 1000 admissions. The total proportion of non-albicans species causing candidemia was 425/618 (69.7%). Overall, 65.4% of candidemia cases occurred in non-critical care units. There was a significant increase in the proportion of *C. auris* isolates between 2016 and 2020 (*p* < 0.001). Isolation of *C. auris* was associated with admission to critical care units (*p* < 0.001, odds ration [OR] 3.856, 95% confidence interval [CI]: 2.360–6.300). The proportion of azole-resistant candidemia cases increased from 21/53 (39.6%) in 2016 to 41/59 (69.5%) in 2020 (*p* = 0.002).

**Conclusion:**

The incidence of candidemia remained stable over the five-year study period. However, the proportion of *C. auris* isolates increased significantly during the study period as did the overall proportion of azole-resistant candidemia. Antifungal stewardship and continued hospital-level surveillance are imperative.

## Introduction

Invasive candidiasis is estimated to have an annual global incidence of 750 000 cases.^1^ It is a condition resulting from medical progress. Based on global estimates of published studies by the Global Action Fund for Fungal Infections (GAFFI), it is ranked fifth amongst the most common life-threatening fungal infections and is associated with an estimated mortality rate of 40%.^1^ A similar study conducted in South Africa ranked candidemia as the top seven most common fungal infections.^2^ The incidence risk of candidemia in South Africa was reported to be 83.8 per 100 000 admissions, with hospital- and species-specific incidence ranging between 0.9 and 375 cases per 100 000 admissions.^3^ Overall, however, there is a paucity of data from the African continent.

Antimicrobial resistance (AMR) has been declared the next great global challenge.^4^ The World Health Organization (WHO) published the Global Action Plan on AMR with specific objectives highlighting the need for antimicrobial development. In addition to the priority bacterial pathogens list published by the WHO, a recent expert group has been formed to create a fungal priority pathogen list for prioritisation of antifungal development.

Coupled with the limited number of antifungal classes for management of fungal infections is the limited access to available antifungal agents in many countries worldwide.^2^ This is becoming increasingly important with the changing epidemiology of candidemia, with species distribution trending towards non-albicans species.^5^ An epidemiological shift towards *Candida parapsilosis* and *Candida auris* has been described in South Africa.^3,6^

There is a paucity of published data describing candidemia at facility level in South Africa. It was the aim of this study to determine the facility-level incidence, species distribution and antifungal resistance of candidemia over a five-year period at a tertiary South African hospital.

## Material and methods

### Study design

A retrospective laboratory-based study was performed from January 2016 to December 2020 using Microbiology Laboratory data at the Charlotte Maxeke Johannesburg Academic Hospital (CMJAH).

#### Setting

Charlotte Maxeke Johannesburg Academic Hospital is a 1088-bed tertiary care hospital located in Johannesburg, South Africa. The hospital provides both general and specialist medical services, including paediatric and adult oncology, paediatric and adult renal and liver transplant, neurosurgery, trauma, neonatal care and cardiothoracic surgery and critical care.

The hospital has a dedicated infection prevention and control unit, and an active antimicrobial stewardship programme (AMS) has been in place since 2017. A 24 hour on-site clinical diagnostic microbiology laboratory services the hospital and includes consultation with specialist clinical microbiologists. In addition to ongoing multidrug resistance (MDR) surveillance by the infection prevention and control department of the hospital, the clinical microbiology department provides a monthly report to hospital management and relevant stakeholders on the incidence of candidemia, species distribution and outbreaks as part of the AMS surveillance activities.

### Study sample

All blood cultures positive with yeast on Gram stain from patients admitted to CMJAH were included. All causative agents of candidemia were included. The database was then deduplicated to include only the first positive culture per pathogen per patient.

### Investigation of suspected candidemia at Charlotte Maxeke Johannesburg Academic Hospital

Unit-specific guidelines for the diagnosis of candidemia were followed. Briefly, this included collection of fungal blood cultures and serum for 1,3-beta-D-glucan (BDG) testing in addition to inflammatory markers and full blood count assessment. Currently, no molecular assays are available in this setting to detect *Candida* species directly from clinical samples. A minimum of one set of blood cultures (BacT/Alert bottles, bioMerieux) would be requested in adult patients, while a single blood culture bottle would be requested for paediatric patients. The bottles were transported to the on-site Microbiology Laboratory and incubated in the BacT/Alert blood culture incubation system. Fungal blood cultures were incubated for 14 days. Processing of positive blood culture bottles occurred 24 h a day. Once a bottle flagged positive, the bottle was removed and a Gram stain performed. If yeasts were observed on the Gram stain, the blood culture broth was subcultured onto 5% sheep blood agar, chocolate agar and Sabouraud Dextrose Agar (SDA) and incubated aerobically for 72 h at 37 °C, with daily observations for growth. Once sufficient pure growth was obtained on the agar plates, identification of the yeast was performed using either the Vitek 2 automated identification and antimicrobial susceptibility testing system or the Vitek MS matrix-assisted laser desorption-time of flight (MALDI-TOF) (bioMerieux, Marcy-L’Etoile), Antifungal susceptibility testing was performed by the Vitek 2 using AST-YS07 and AST-YS08 cards or gradient diffusion strips. Antifungal susceptibility was interpreted using the Clinical Laboratory Science Institute (CLSI) M27A3.

*Candida auris* identification was only performed in the laboratory from October 2017. Prior to this, suspected *C. auris* isolates were submitted to the national reference laboratory for identification. *Candida auris* was suspected if *Candida haemulonii* or *Candida famata* were identified or any *Candida* species with unexpected high minimum inhibitory concentrations (MICs) to azoles.

All positive cultures with yeasts were communicated to the attending clinician or to a clinical member of staff in the unit telephonically or as a bedside consultation by the microbiologist. Identification and antifungal susceptibility result were also communicated as they became available.

### Management of suspected candidemia at Charlotte Maxeke Johannesburg Academic Hospital

Based on national surveillance data prior to 2015 demonstrating a shift in epidemiology of candidemia to non-albicans species, and the limited availability of antifungal agents, recommendations for empiric management of candidemia at CMJAH were changed from fluconazole to Amphotericin B. These recommendations were communicated to clinicians through a monthly candidaemia report detailing the species distribution throughout the hospital. Amphotericin B was used because liposomal Amphotericin and Amphotericin lipid formulation are not available in the South African public health sector. De-escalation to fluconazole was recommended based on the identified species and antifungal susceptibility results combined with documented clearance from blood culture and adequate source control. Micafungin became available in 2017 for cases with confirmed resistance to azoles/amphotericin B, or in patients with severe renal dysfunction.

Not all patients with confirmed candidemia received infectious diseases (ID) consultation as a result of a shortage of qualified ID clinicians at the hospital. Only a single qualified ID physician and no ID paediatricians were available during the study period. Certain departments had the expertise of other specialists with special interest in ID who were knowledgeable and experienced in the management of candidaemia. In other clinical departments, recommendations for appropriate clinical management and antifungal stewardship were also an integral function of the clinical microbiologists.

### Data collection

Laboratory data were extracted from the laboratory information system. The extracted data included age, gender, clinical department, species and antifungal susceptibility results. Monthly hospital admission data were acquired from the hospital administrator. Polymicrobial bloodstream infections including species causing candidemia and bacterial pathogens were included. Reinfection, new infections and persistence could not be determined because of the lack of clinical information collected.

Individual patient-isolate cases were stratified based on ward type – critical care versus non-critical care – and based on the clinical department (medical, surgical, neonatal, oncology, paediatrics and transplant).

Isolates were classified as MDR or extensively drug resistant (XDR) based on the definitions suggested by Arendrup and Patterson.^7^ Multidrug resistant was defined as an isolate non-susceptible to ≥ 1 agent in ≥ 2 drug classes and XDR was defined as an isolate non-susceptible to ≥ 1 agent in ≥ 3 drug classes.

### Data and statistical analysis

Initial data analysis was performed in Microsoft Excel and statistical analysis was performed using Statistical Package for the Social Sciences (SPSS) Statistica version 25 and Epitools. Candidemia incidence was calculated per 1000 admissions. Descriptive data were reported as frequencies and percentages. Kendall’s tau-b correlation was used to determine the relationship between time and candidemia incidence. Chi-squared test was used to compare demographic features per species. Two-sided *p*-values were used consistently. Where large adjusted standardised residuals were noted, these were further analysed as binary variables to determine the odds ratio (OR) of intensive care unit (ICU) versus non-ICU species causing candidemia. Chi-squared Fisher exact test using weighted cases procedure was used to compare species and ward type. Two-sample *Z*-test was used to determine differences in proportion of isolates per species between 2015 and 2020.

### Ethical considerations

Permission to conduct the study was obtained from the Human Research Ethics Committee (HREC) of the University of the Witwatersrand, (clearance number: M180625) as well as from hospital management.

## Results

**FIGURE 1:**
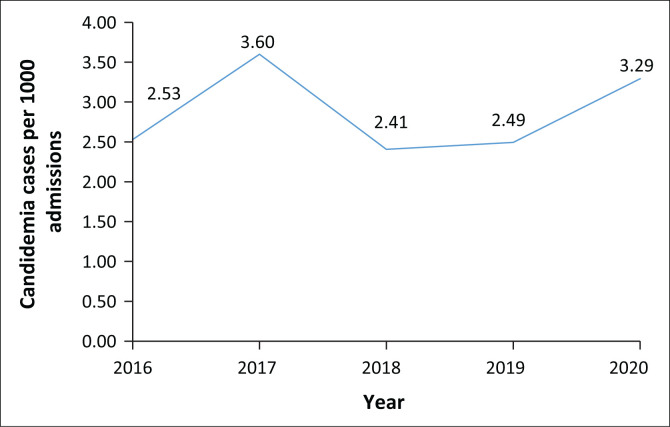
Trends in candidemia incidence per 1000 admissions 2016–2020.

From 2016 to 2020, a total of 19 923 deduplicated positive blood culture isolates were identified. When common commensal organisms were removed from the list, species causing candidemia ranked among the top six bloodstream infection causing pathogens. The overall incidence of candidemia during the study period was 2.87 per 1000 admissions, with some variability each year. Figure 1 demonstrates the yearly incidence of candidemia. A Kendall’s tau-b correlation was run to determine the relationship between time and candidemia incidence. No significant association was found between time and candidemia incidence.

**FIGURE 2:**
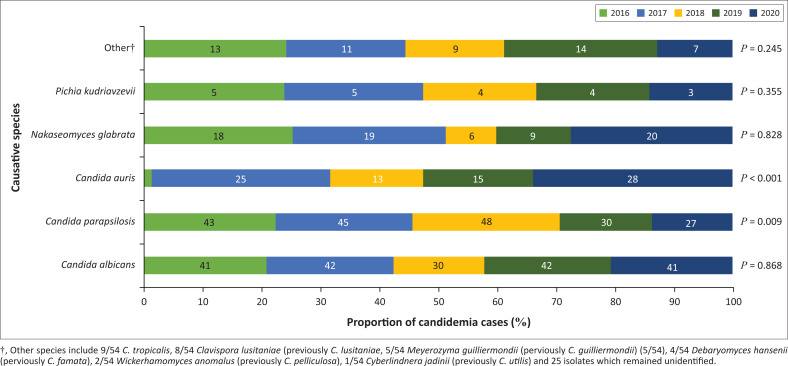
Trends in yearly species distribution, 2016–2020.

During the study period, 618 isolates were identified from 610 patients, with 196/618 (31.72%) *Candida albicans*, 193/618 (31.23%) *C. parapsilosis*, 82/618 (13.27%) *C. auris*, 72/618 (11.65%) *Nakaseomyces glabrata* (previously *Candida glabrata*), 21/618 (3.40%) *Pichia kudriavzevii* (previously *Candida krusei*) and 54/618 (8.74%) other or unidentified species isolated from blood cultures. Of the 610 cases, 211 (34.6%) occurred in patients in critical care units. Proportion of cases from the various clinical departments was as follows: 213/610 (34.9%) from neonatal department, 135/610 (22.1%) in the surgical department, 130/610 (21.3%) in the medical department, 66/610 (10.8%) from the paediatric department, 64/610 (10.4%) from the oncology department and 2/610 (0.35) from the Transplant unit. Figure 2 demonstrates the temporal distribution per species and the difference in proportion each species contributed in 2016 compared to 2020.

Patient demographics, level of care (critical care vs. non-critical care) and hospital department, representative of broader underlying condition per species, are represented in Table 1. Frequency of *C. auris* isolation from critical care units was greater than non-critical care units when analysed per species (*p* < 0.001, OR: 3.856, 95% confidence interval [CI]: 2.360–6.300). During comparison of each species to ward type, using chi-squared test with weighted cases procedure, a comparison of *C. albicans* to all non-albicans species found the frequency of *C. albicans* isolation from medical wards to be greater compared to other wards (*p* = 0.004, OR: 1.827, 95% CI: 1.226–2.273).

**TABLE 1:** Patient demographics, level of care and clinical department per species.

Variable	*Candida albicans*(*N* = 196)		*Candida auris*(*N* = 82)		*Nakaseomyces glabrata* (*N* = 72)		*Pichia kudriavzevii*(*N* = 21)		*Candida parapsilosis* (*N* = 193)		Other species (*N* = 54)		*p* (chi square)
	*n*	%	*n*	%	*n*	%	*n*	%	*n*	%	*n*	%	
Female sex	89	45.0	37	45.1	38	52.7	14	66.7	92	47.7	24	44.4	0.328
Critical care	59	30.1	52	63.4	25	34.7	7	33.3	55	28.5	13	24.1	0.001
Medical (*N* = 130)	56	28.6	22	26.8	25	34.8	4	19.1	15	7.8	8	14.9	0.001
Neonatal (*N* = 213)	61	31.1	14	17.1	11	15.3	3	14.3	109	56.5	15	27.8	-
Oncology (*N* = 64)	20	10.2	1	1.2	5	6.9	7	33.3	21	10.9	10	18.5	-
Paediatrics (*N* = 66)	28	14.3	1	1.2	5	6.9	3	14.3	24	12.4	5	9.3	-
Surgical (*N* = 135)	31	15.8	41	50.0	25	34.7	3	14.3	24	12.4	11	20.4	-
Transplant (*N* = 2)	0	-	0	-	1	1.4	0	-	0		1	1.9	*

In order to determine the difference between species and ward type, further post hoc analysis of the chi-square contingency table showed significant differences between all species, across all wards types, the following significant differences were noted: there was a significant difference between *C. auris* and non-*C. auris* species in neonates versus non-neonates (*p* < 0.001, OR: 0.357, [0.195–0.653]), surgical wards versus non-surgical wards (*p* < 0.001, OR: 0.200, [0.122–0.328]), and oncology wards versus non-oncology wards (*p* = 0.01, OR: 10.545, [1.442–77.139]), but no difference between medical and non-medical wards (*p* = 0.142).

Based on available automated antifungal susceptibility testing results during the study period, 1/426 (0.2%) *C. albicans* and 201/426 (47.2%) non-albicans isolates were resistant to triazoles; 1/491 (0.2%) *C. albicans* and 4/491 (0.9%) non-albicans isolates were resistant to amphotericin; 1/363 (0.3%) *C. albicans* and 24/363(6.6%) non-albicans isolates were resistant to echinocandins and 11/403 (2.5%) *C. albicans* and 18/403 (4.5%) non-albicans isolates were resistant to flucytosine. Fourteen isolates were categorised as multidrug resistant; 1/14 *C. auris*, 2/14 *C. parapsilosis* and 11/14 *Pichia kudriavzevii*. The proportion of azole-resistant isolates increased from 21/53 (39.6%) in 2016 to 41/59 (69.5%) in 2020 (*p* = 0.002).

## Discussion

This was a five-year retrospective review of the incidence, distribution and antifungal susceptibility patterns of hospital-wide candidemia at a tertiary South African hospital. We examined trends over the five-year period. The available published candidemia-related literature from South Africa consists of national surveillance data or unit-specific data. This is the first study, to our knowledge, which examines candidemia at hospital level in South Africa.

Our data demonstrate an incidence of 2.87 cases of candidemia per 1000 admissions, ranging from 2.41 to 3.60. The incidence risk for candidemia was reported as 83 cases per 100 000 admissions from a national surveillance study.^3^ Population-specific incidences from South Africa include reported incidences of 2.8 per 10 000 adult admissions from a study conducted in 1990–2007 to 5.3 cases per 1000 paediatric admissions at tertiary hospitals.^8,9^ However, as a result of non-standardised reporting of incidence, no meaningful assessments can be made.

Although the highest overall individual number of isolates in our study was *C. albicans* (196/618; 31.72%), there was almost an equal number of *C. parapsilosis* isolates (193/618; 31.23%). Recently published national surveillance data found *C. parapsilosis* to be the predominant species causing candidemia in South Africa and a shift in epidemiology caused by the emergence of *C. auris.*^3^ Our study also demonstrates the prominent role of *C. auris* during the study period with a significant increase in proportion of *C. auris* isolates between 2016 and 2020. The location of CMJAH in Gauteng province is also consistent with the national surveillance data findings. Of interest is the significant reduction in proportion of *C. parapsilosis* isolates from 2016 to 2020. This finding requires continuous monitoring to determine if it represents a true change or an incidental finding. The introduction of *C. auris* into this setting may result in species replacement over *C. parapsilosis* if appropriate infection prevention and control measurements are not adhered to. *Candida auris* is known to survive harsh environmental stressors and its environmental biofilms facilitate nosocomial transmission.^10^

Candidemia is typically associated with admission to ICUs. In our study, patients in critical care units were more likely to culture *C. auris* from blood cultures compared to other species.^11^ The remainder of species causing candidemia in our study were isolated more commonly in non-critical care units. The predominance of non-albicans species in clinical departments other than the medical department is also noteworthy. Overuse or misuse of antifungal agents as prophylaxis and broad spectrum antibacterial agent use which may be driving the predominance of non-albicans species in these units requires further investigation.

Antifungal susceptibility results in our study demonstrate a predominance of non-albicans azole resistance. Prior antibiotic exposure has been noted to be a risk factor for fluconazole-resistant bloodstream infections.^12^ High rates of AMR at our institution have resulted in broad spectrum antimicrobials being used as empiric therapy.^13,14^ The statistically significant increase in azole resistance over the five-year study period is of concern. We have previously published data from our laboratory demonstrating the variable sensitivity of the BDG assay for detecting different species causing candidemia. It is our view that this assay, in the absence of more accurate, sensitive and specific diagnostic tests for candidemia, may perhaps be used inappropriately in certain units, both with low pre-test probability of candidemia and inappropriate treatment based on low positive BDG results. However, prospective clinical studies which include the value of other biomarkers such as C-reactive protein (CRP) and procalcitonin (PCT) are required in our setting to validate this theory.

The proportion of cases which are MDR is likely not accurate because the reference method of broth microdilution was not used in our study. In addition, the Vitek 2 is unable to report interpretations for antifungal agents in the absence of clinical breakpoints. Thus, results for those isolates were not in our database. Publications reporting rates of MDR candidemia mainly focus on *C. auris* and *N. glabrata.*^7^ With rising rates of AMR globally, the WHO has convened a panel of antifungal experts to compile a list of fungal priority pathogens to aid development of antifungal agents.^15^ Standard definitions of MDR and XDR for candidemia are essential, as is the need for studies reporting on patterns of multi and extensive drug resistance amongst candidemia causing pathogens.

Numerous publications have described increased incidence of candidaemia in COVID-19 patients.^16,17,18^ The effect of the COVID-19 pandemic on candidaemia incidence at this institution requires additional investigation. The upward trend in incidence noted in 2020 may well be the result of the pandemic. However, further clinical case control studies are required to verify this relationship.

There is a paucity of data from the African continent by which we could benchmark our hospital-level findings. A multicentre surveillance study in Algeria found *Candida tropicalis* to be the most prevalent species causing candidemia.^19^ Thirty-eight per cent of candidemia cases at a single centre in Kenya were found to be because of *C. auris*, with *C. albicans* accounting for only 25% of cases.^20^ An intensive care outbreak of *Yarrowia lipolytica* candidemia was reported from a single centre in Tunisia.^21^ A retrospective study conducted at a neonatal unit in Lagos, Nigeria demonstrated a predominance of *C. albicans* causing neonatal candidemia.^22^ Further hospital-level data from the continent are needed to highlight the burden of candidemia in Africa.

## Limitations

Our study has a number of limitations. The lack of clinical information, and treatment and outcome data is a major limitation of our study which restricts the clinical impact of the data we present. Additional clinical information would have provided invaluable information and associations regarding the pathogens identified and the candidemia itself. Age, immune status, presence of malignancy and type of malignancy, surgical procedures performed, the presence of intravascular catheters, prior use of antibiotics, corticosteroid or other immunosuppressive therapy, severity of illness scores, life support devices and other interventions such as mechanical ventilation and renal replacement therapy, length of hospitalisation and outcome would enhance the nature of the data presented here. The absence of an electronic dispensing system prevented analysis of antifungal drug doses prescribed and dispensed. This also prevented details regarding antifungal prophylaxis use in the institution from being collected. These would have provided additional insight into the epidemiology noted. The retrospective nature of a single-centre study also prevents this data from being generalised on a broader scale. Lack of clinical follow-up prevented inclusion of repeat episodes of candidemia by the same species from being included in the database. This lack of clinical follow-up also prevented accurate classification of the infection based on clinical unit type (e.g. critical care vs. non-critical care). MIC values as reported by the Vitek 2 were not used for analysis – only the interpretation was included. Advanced expert system analysis on the Vitek 2 may have resulted in MIC interpretations which differ from the reported MIC value. Being a retrospective review, antifungal susceptibility results could not be checked. This is particularly relevant for the single case of echinocandin-resistant *C. albicans* noted. Outbreaks and clusters of cases were not investigated as part of this study. The number of unidentified species may have resulted in under-reporting of isolates such as *C. auris* which is commonly misidentified by automated identification systems. As mentioned previously, under-reporting of MDR is likely as a result of the non-reporting of antifungal susceptibility results for *C. auris* by the Vitek 2.

However, our study has highlighted a number of important findings which will allow for future endeavours. Ongoing hospital-based surveillance, including reporting of incidence and species distribution alongside clinician education, is important to ensure appropriate management of candidemia at CMJAH. Diagnostic stewardship measures to optimise the use of the BDG assay are necessary to ensure that the test is ordered in patients at risk of candidemia and to ensure that results are not interpreted inappropriately in the absence of additional investigations. Follow-up clinical studies are required in our setting to determine the risk factors and outcomes associated with infection. Antimicrobial stewardship activities and specific antifungal stewardship activities targeting inappropriate antifungal prescribing must be prioritised in order to limit the rising rates of azole-resistant candidemia. Further hospital-level studies from the African continent are required to determine the true burden of disease caused by candidemia and to further improve diagnostics, management and infection prevention and control measures on the continent.

## Conclusion

In summary, our findings mirror the changing epidemiology of candidemia globally. Increasing prevalence of azole-resistant candidemia is of particular concern. The WHO’s Global Action plan on AMR specifies surveillance and appropriate antimicrobial use as two main objectives to combat AMR. Hospital-level surveillance for candidemia should be an integral aspect of hospital-based AMS programmes and include monitoring of incidence, species distribution and antifungal susceptibility patterns. Where national surveillance systems are not feasible, hospital-level surveillance may provide a glimpse of regional candidemia epidemiology.
